# Involvement of bacterial TonB-dependent signaling in the generation of an oligogalacturonide damage-associated molecular pattern from plant cell walls exposed to *Xanthomonas campestris* pv. campestris pectate lyases

**DOI:** 10.1186/1471-2180-12-239

**Published:** 2012-10-19

**Authors:** Frank-Jörg Vorhölter, Heinrich-Günter Wiggerich, Heiko Scheidle, Kalina Mrozek, Alfred Pühler, Karsten Niehaus

**Affiliations:** 1 Department of Proteome and Metabolome Research, Faculty of Biology, Universität Bielefeld, Universitätsstr. 25, Bielefeld, 33615, Germany; 2 CeBiTec, Universität Bielefeld, Universitätsstr. 27, Bielefeld, 33615, Germany; 3 Laboratory of Molecular Signaling, 5625 Fishers Lane, National Institute on Alcohol Abuse and Alcoholism, National Institutes of Health, Rockville, MD, 20852, USA; 4 Institut für Pflanzengenetik, Naturwissenschaftliche Fakultät, Leibniz Universität Hannover, Herrenhäuser Str. 2, Hannover, 30419, Germany

**Keywords:** TonB system, Damage-associate molecular pattern, DAMP, Oligogalacturonide, Trans-envelope signaling, Molecular plant-microbe interaction, Pathogen, Xanthomonas campestris

## Abstract

**Background:**

Efficient perception of attacking pathogens is essential for plants. Plant defense is evoked by molecules termed elicitors. Endogenous elicitors or damage-associated molecular patterns (DAMPs) originate from plant materials upon injury or pathogen activity. While there are comparably well-characterized examples for DAMPs, often oligogalacturonides (OGAs), generated by the activity of fungal pathogens, endogenous elicitors evoked by bacterial pathogens have been rarely described. In particular, the signal perception and transduction processes involved in DAMP generation are poorly characterized.

**Results:**

A mutant strain of the phytopathogenic bacterium *Xanthomonas campestris* pv. campestris deficient in *exbD2*, which encodes a component of its unusual elaborate TonB system, had impaired pectate lyase activity and caused no visible symptoms for defense on the non-host plant pepper (*Capsicum annuum*). A co-incubation of *X. campestris* pv. campestris with isolated cell wall material from *C. annuum* led to the release of compounds which induced an oxidative burst in cell suspension cultures of the non-host plant. Lipopolysaccharides and proteins were ruled out as elicitors by polymyxin B and heat treatment, respectively. After hydrolysis with trifluoroacetic acid and subsequent HPAE chromatography, the elicitor preparation contained galacturonic acid, the monosaccharide constituent of pectate. OGAs were isolated from this crude elicitor preparation by HPAEC and tested for their biological activity. While small OGAs were unable to induce an oxidative burst, the elicitor activity in cell suspension cultures of the non-host plants tobacco and pepper increased with the degree of polymerization (DP). Maximal elicitor activity was observed for DPs exceeding 8. In contrast to the *X. campestris* pv. campestris wild type B100, the *exbD2* mutant was unable to generate elicitor activity from plant cell wall material or from pectin.

**Conclusions:**

To our knowledge, this is the second report on a DAMP generated by bacterial features. The generation of the OGA elicitor is embedded in a complex exchange of signals within the framework of the plant-microbe interaction of *C. annuum* and *X. campestris* pv. campestris. The bacterial TonB-system is essential for the substrate-induced generation of extracellular pectate lyase activity. This is the first demonstration that a TonB-system is involved in bacterial trans-envelope signaling in the context of a pathogenic interaction with a plant.

## Background

Plant cells are permanently monitoring their immediate environment to identify attacking pathogens and subsequently initiate defense. Highly conserved molecular structures termed microbe-associated molecular patterns (MAMPs) or pathogen-associated molecular patterns (PAMPs) are obvious targets for this recognition process. The term PAMP-triggered immunity (PTI) is increasingly used for this innate immunity [1]. Recognition by the plant employs transmembrane pattern recognition receptors (PRRs). Unfortunately, so far there are only a few detailed model systems that describe MAMP, PRR, and perception-induced signaling [2]. An example for such a well-characterized PTI is the recognition of bacterial flagellin in *Arabidopsis thaliana*[3]. In older literature, molecules which evoke defense-related plant reactions and which hence are assumed to be involved in the recognition process of non-host plants were termed elicitors [2]. Plant defense upon pathogen recognition typically includes the induction of a so-called hypersensitive response (HR), which leads to the resistance of the non-host plants and which includes a rapid local generation of superoxide, the so-called *oxidative burst*, and a programmed cell death [4]. Examples for MAMPs are the harpin proteins from *Erwinia*[5,6], *Xanthomonas*[7,8], or *Pseudomonas*[9], syringolides from *Pseudomonas syringae*[10] or lipopolysaccharides (LPSs), characteristic glycoconjugate cell envelope constituents of Gram-negative bacteria [11].

In addition to monitoring for pathogen-derived MAMPs, plants recognize endogenous molecules that are released upon injury or infection as alarm signals. Such molecules are termed damage-associated molecular patterns (DAMPs) [12]. Often DAMPs are generated by lytic enzymes of attacking pathogens when they breach structural barriers of plant tissues, in particular plant cell walls. DAMPs include oligosaccharide fragments, peptides resulting from protein degradation [13], and reactive oxygen species (ROS) [14]. Plants can amplify the response to DAMPs by inducing specific enzymes that generate additional similar DAMP molecules [15]. Examples for DAMPs known for a long time are oligogalacturonides (OGAs) that are released by fungal pectate lyases [16-18] from plant cell walls. Among the plant pathogenic bacteria, so far only *Erwinia carotovora* has been reported to induce the generation of a DAMP [19], which also turned out to be an OGA [20]. Upon the discovery of the egg box conformation of OGA dimers [21], the *A. thaliana* wall-associated kinase 1 (WAK1) was identified as a candidate for a PRR that specifically recognizes OGAs. While the receptor-like kinase WAK2 was shown to be involved in pectin-dependent signaling [22], a recent domain-swap experiment confirmed the identification of WAK1 as OGA receptor [23], thereby turning the plant side of OGA perception into a comparably complete model of DAMP recognition.

*Xanthomonas* species are members of the γ subdivision of the Gram-negative *Proteobacteria*, which have adopted a plant-associated and usually plant pathogenic lifestyle [24,25]. *Xanthomonas campestris* pv. campestris is a pathogen of *Brassicacea* including *A. thaliana*. Upon infection of cabbage plants it causes the black rot disease. In non-host plants like pepper (*Capsicum annuum*) and tobacco (*Nicotiana tabacum*), however, it induces an HR. For *X. campestris* pv. campestris, LPSs [26-29], as well as muropeptides [30], fragments of the bacterial cell wall material peptidoglycan, have been characterized as MAMPs. Non-host resistance of plants towards *X. campestris* pv. campestris seems to be a very complex situation, where multiple elicitors are active in parallel [26,31]. The genetic analyses performed during the last years identified several gene loci that are linked to the pathogenicity of *X. campestris* pv. campestris in host plants and to the induction of a resistance response in non-host plants. Protein secretion systems, in particular the type III secretion system, have an important role in the pathogenic interactions with plants [32-35]. Further virulence factors are exported by type II secretion systems [32,36]. They are involved in the secretion of extracellular enzymes including plant cell wall degrading enzymes like pectate lyases (EC 4.2.2.2), also known as polygalacturonate lyases [37-40], or polygalacturonases (EC 3.2.1.15) [40,41]. Pectate lyases catalyze the cleavage of α­1,4 glycosidic bonds between galacturonic acid residues of homogalacturonans. Likewise, polygalacturonases catalyze the cleavage of the glycosidic bonds between adjacent galacturonic acid residues, but the hydrolysis of the glycosidic linkage results in the addition of a water molecule from the environment. Genome data which are now available for several strains have further added to our understanding of pathogenicity loci in *X. campestris*[42-47]. More information can be derived from closely related pathogens like *Xylella fastidiosa*, where a polygalacturonase has been characterized that is similar to the *pglA2* gene product of *X. campestris* pv. campestris B100 [48]. Rapid progress is currently achieved in identifying and analyzing regulation in *X. campestris*[49-52]. Concerning signal transduction, there has been substantial advancement of science related to two complex systems of cell-cell communication that employ a diffusible signal factor (DSF) [53] and a diffusible factor (DF) [54], respectively. In addition, more and more *X. campestris* two-component systems signal-transduction systems are characterized experimentally [55-58].

In previous analyses, the *X. campestris* pv. campestris *tonB* gene cluster showed some very interesting characteristics. TonB systems of Gram-negative bacteria are multi-component transport systems that perform the specific active uptake of various compounds across the outer membrane [59]. These systems consist of the core components TonB, ExbB, and ExbD, which are located at or within the inner membrane, and variable so-called TonB-dependent receptors, which are located in the outer membrane, and which are specific for the imported substrate [60]. TonB is assumed to transfer energy, which is gathered by the inner membrane components ExbB and ExbD from the inner membrane’s proton motive force, to the TonB-dependent receptors in the outer membrane. The TonB system is particular known for the uptake of iron [61]. For *X. campestris* pv. campestris, an unusual high number of diverse TonB-dependent receptors has been identified in a profound analysis [62]. Functional data revealed, besides iron, carbohydrates as substrates imported by specific TonB-dependent receptors of *X. campestris* pv. campestris [62]. A gene of a TonB-dependent receptor that was co-located wtih genes for two putative pectin/polygalacturonate degrading enzymes was induced by polygalacturonate [62]. TonB-dependent receptors are part of a regulon involved in utilization of *N*-acetylglucosamine, but their specific role remained unclear [63]. The contiguous *X. campestris* pv. campestris genes *tonB*, *exbB*, and *exbD1,* which code for the TonB system core components, are essential for iron uptake [64]. They are also required to induce the black rot disease in *Brassica oleracea*, to induce an HR in the interaction with the non-host plant *C. annuum*, and they are involved in the infection of *X. campestris* pv. campestris by the lytic bacteriophage ΦL7 [65]. Differing from other Gram-negative bacteria, in *X. campestris* pv. campestris there is a similar second *exbD* gene, termed *exbD2*, which is located in the same gene cluster *in tandem* directly downstream of *exbD1*[64]. This gene is not essential for iron-uptake, not necessary to induce the black rot symptoms on host plants, and not essential for penetration by phage ΦL7, but it is required to induce an HR in non-host plants [66]. A similar but not identical genetic organization with two *exbD* genes located *in tandem* has only been described for the fish-pathogenic *Flavobacterium psychrophilum*, where again the *exbD2* gene, which was also not required for iron uptake, was involved in pathogenicity [67]. Although the role of the *X. campestris* pv. campestris *exbD2* gene is not well understood in detail, there are hints that the gene product is involved in the export of *X. campestris* pv. campestris exoenzymes.

In this study, we have analyzed the *exbD2* gene in more detail. In the course of the analyses, we discovered that *exbD2* is involved in the induction of bacterial pectate lyase activity, which then releases OGAs from plant-derived pectate that are subsequently recognized as a DAMP by the plant.

## Results

The structure of the *tonB* gene cluster of *X. campestris pv.* campestris is unusual, and the role of the second *exbD* gene located in this cluster is still puzzling. Differing from the genes *tonB*, *exbB*, and *exbD1*, *exbD2* is not required for iron uptake [64], but it is essential to induce an HR on *C. annuum*[66]. Hence, further analyses were performed to obtain a better understanding of this enigmatic pathogenicity-related gene.

### Genomic analysis of *X. campestris* pv. campestris TonB core genes

**Figure 1:**
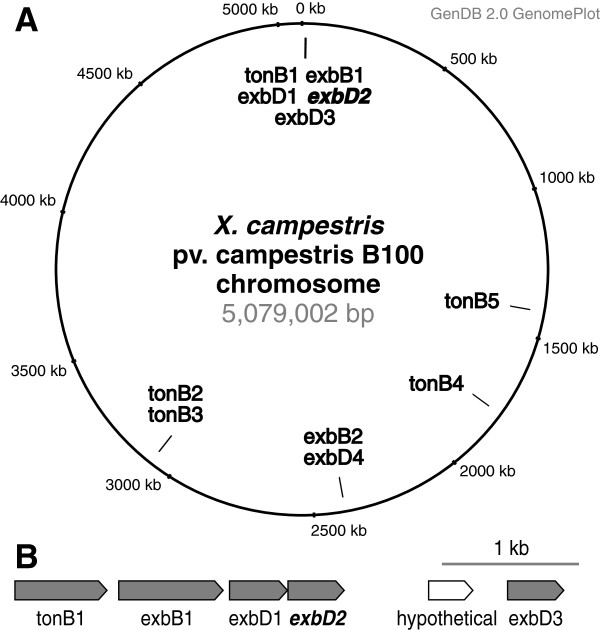
**Genomic organization of the TonB-related genes in *X. campestris *pv. campestris B100.** (**A**) A circular genome plot indicates the locations of the TonB-related genes on the chromosome. The core of the TonB system is encoded by the genes *tonB*, *exbB* and *exbD*. In *X. campestris* pv. campestris B100 multiple isoforms of these genes were identified. Their genomic locations on the circular chromosome are indicated. So far, this multiplicity was only known for *tonB* genes in *Pseudomonas*[68] and for the *exbD* genes in *Flavobacterium psychrophilum*, where two paralogous genes were found in tandem in a cluster combined with *tonB* and *exbB*[64] close to the chromosomal origin of replication (**B**). Size and direction of transcription is illustrated by arrows for this gene cluster. Genes that were predicted with convincing evidence are symbolized by shaded arrows, while an open arrow indicates a putative protein-coding sequence (CDS) that was predicted with less confidence. Now a third copy of *exbD* was found downstream of *exbD2*, separated from *exbD2* only by a hypothetical gene for which nor functionality neither expression could be indicated. Further copies of *tonB* and the genes *exbB-exbD* were found at different chromosomal positions. To facilitate discriminating the individual genes, unique numbers were added to their names.

The availability of genome data [43,45,46] allowed a broader perspective on the TonB system of *X. campestris pv.* campestris. This led already to the discovery of an unexpected wealth of TonB-dependent receptors [62]. A detailed genomic analysis revealed now the presence of further genes coding for components of TonB systems (Figure 1A). In total, five copies of *tonB*, two copies of *exbB* and four copies of *exbD* were identified within the genome. Downstream of the previously characterized *tonB-exbB-exbD1-exbD2* genes, which are located close to the chromosomal origin of replication, a third *exbD* gene was identified (Figure 1B). While the presence of different TonB-dependent receptors has been attributed to their distinct binding specificities, where different molecules are bound at the outer cell surface to be either transported inside or to signal their presence to the cell interior, so far it has been assumed that only one set of *tonB-exbB-exbD* genes is required to build a TonB protein complex that interacts with all the different TonB-dependent receptors. Results of previous mutational analyses [64] suggest that the newly identified genes of TonB system core components are not involved in iron uptake. To shed more light on the multiplicity of these genes, we concentrated on analyzing the function of *exbD2*, which had already been shown to be involved in plant interaction, despite being not important for iron uptake [66]. A genomic comparison showed that this gene was present and well conserved in all complete *Xanthomonas* genomes (Additional file 1).

### The *exbD2* gene is involved in pectate lyase activity

**Figure 2:**
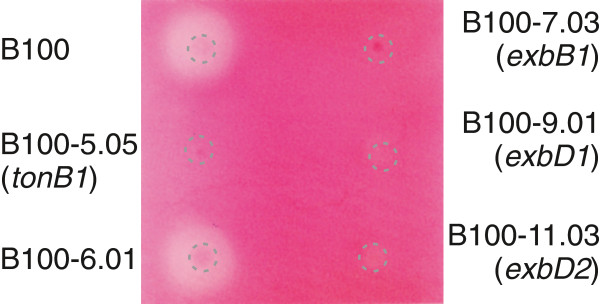
**Test for pectate lyase activity in TonB-related mutants of *X. campestris *pv. campestris.***X. campestris* pv. campestris wild-type strain B100 and mutants derived from it with disrupted genes coding for core components of the TonB system were grown for two days on M9 minimal medium supplemented with pectate and FeSO_4_. The positions of the inocula are indicated by dashed circles. Staining with Ruthenium Red unveiled halos encircling the inocula of the wild-type and a control strain that indicate activity of extracellular pectate lyases [64], while no halos were visible when the genes *tonB1*, *exbB1*, *exbD1*, and *exbD2* were disrupted. The mutant strain B100-6.01 [64], carrying an ΩKm(*cat*) insertion in the non-coding region between *tonB* and *exbB*, was tested as a positive contro.

*X. campestris pv.* campestris is well-known for its pathogenicity-related extracellular enzymes, among them a pectate lyase [37,38]. Mutant strains affected by insertions of ΩKm(*cat*) cassettes in *tonB1*, *exbB1*, *exbD1*, and *exbD2*[64] were not reduced in the activities of their extracellular amylases, proteases, and carboxymethyl cellulases, respectively, when compared to the wild-type (data not shown). However, an agar plate test for pectate lyase activity showed no activity for mutants deficient in *tonB1*, *exbB1*, *exbD1*, and *exbD2*, while there were substantial halos caused by pectate degradation around colonies of the wild-type and a positive control (Figure 2). The lost extracellular pectate lyase activity could be recovered for all mutant strains including the *exbD2* mutant B100-11.03 by introducing plasmids carrying specific copies of the complete genes [64,66]. The halos encircling the complemented mutants were only slightly less pronounced in size than halos around the wild-type strain B100 (Additional file 2). Due to the parallel presence of genes for pectate lyases and polygalacturonases in *X. campestris pv.* campestris B100, it is in several cases impossible to distinguish between these enzyme classes by means of phenotypic effects such as digestion of polygalacturonic acid in agar plate tests. In such cases, the term "pectate lyase" is used in a loose manner in this manuscript and meant to include polygalacturonases.

These first results were checked in a more elaborate approach. The strains B100-5.05 (*tonB1*), B100-7.03 (*exbB1*), B100-9.01 (*exbD1*), B100-11.03 (*exbD2*), and the wild-type were grown in liquid medium under inducing conditions. The pectate lyase activity was determined in a photometric assay [38]. In contrast to the wild-type, all mutant samples showed no pectate lyase activity, see Additional file 3: Table S1. As no structural genes coding for pectate lyase enzymes were affected by the *X. campestris* pv. campestris mutations analyzed, it seemed likely that the mutations in the genes *tonB1*, *exbB1*, *exbD1, and exbD2* affected the induction of pectate lyase genes.

### Pectate lyase activity is required for HR on *C. annuum*

**Figure 3:**
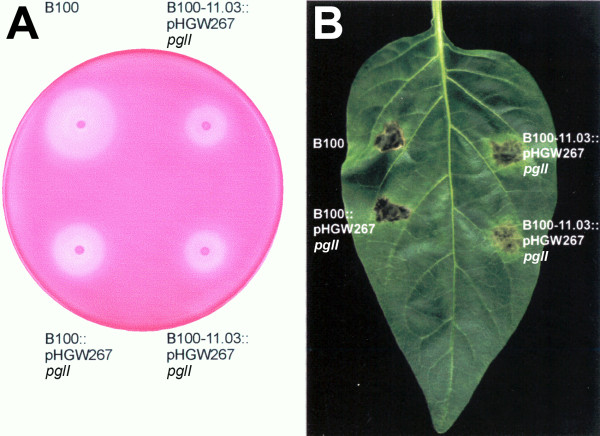
**Complementation of an *X. campestris *pv. campestris *exbD2 *mutant by a constitutively expressed *pglI *gene from *X. campestris *pv. campestris 8004.** When compared to the *X. campestris* pv. campestris wild-type strain B100, it becomes obvious that the mutant strain defective in *exbD2*, B100-11.03, which had been demonstrated before to induce no symptoms like necrotic lesions [66], could be functionally complemented with a constitutively expressed *pglI* gene on plasmid pHGW267 that was integrated into the chromosome. (**A**) The complemented mutant strain regained its pectate lyase activity, although not to the full extent of the wild-type strain. (**B**) This correlates well with the reconstituted but attenuated hypersensitive response that this complemented mutant evoked on *C. annuum.*

Since the breakdown of pectate in plant cell walls is supposed to be an important factor in the pathogenic interaction of *X. campestris* pv. campestris with its host plants, the missing pectate lyase activity could be a reason for the absence of HR in the *X. campestris* pv. campestris mutants defective in *tonB1*, *exbB1*, *exbD1*, or *exbD2*. This hypothesis was checked in a complementation experiment. The *pglI* gene coding for pectate lyase isoform I had been functionally characterized based on *X. campestris* pv. campestris wild-type strain 8004 [38,39]. This gene, which is orthologous to the *X. campestris* pv. campestris B100 gene termed *pel1*, was cloned from cosmid pIJ3051 [39] to finally obtain the plasmid pHGW267, where *pglI* was constitutively expressed under the control of the *aacC1* P_out_ promoter (see methods section for details). This plasmid, which could not replicate in *X. campestris* pv. campestris, was integrated into the chromosomes of the *X. campestris* pv. campestris wild-type strain B100 and of the *exbD2* mutant, which was not affected in iron uptake [64]. The pectate lyase of the resulting complemented strains was also active in the absence of pectate, although the activity was decreased by about 50% when compared to the pectate-induced wild-type (Additional file 3: Table S2). So these strains did not require induction for pectate lyase activity. Both *X. campestris* pv. campestris strains carrying the constitutively expressed *pglI* gene, the wild-type as well as the *exbD2* mutant, were then infiltrated into *C. annuum* leafs. Here, the complemented *exbD2* mutant induced an HR with symptoms similar to the wild-type, although with a delay of one day (Figure 3). Hence, the intensity of the HR correlated well with pectate lyase activity. The results show that *X. campestris* pv. campestris pectate lyase activity is required to invoke an HR on pepper.

### Elicitor-activity upon co-incubation of *X. campestris* pv. campestris with *C. annuum* cell wall material

The successful complementation of an *exbD2* mutant with a pectate lyase gene indicated an important role of this gene in the recognition of *X. campestris* pv. campestris pathogens by non-host plants. However, the molecular characteristics of the elicitor that caused the HR were still unknown. The pectate lyase itself could act as a MAMP. Alternatively, products of the pectate lyase reaction like depolymerized pectate fragments derived from the plant cell wall could be recognized by the non-host plants. So, also a DAMP could not be ruled out as a possible cause of the HR.

**Figure 4:**
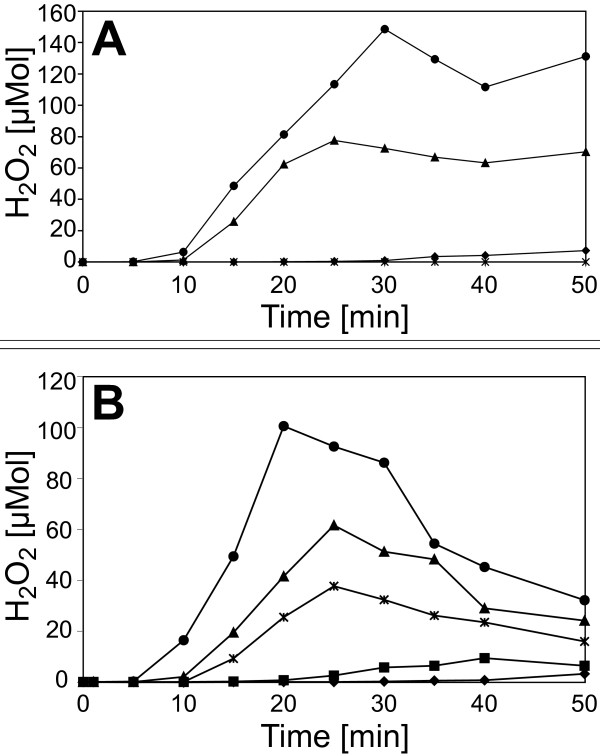
***Oxidative burst *reactions in heterologous *N. tabacum *cell suspension cultures after elicitation with supernatants of *X. campestris *pv. campestris co-incubated with plant cell wall material.** The production of hydrogen peroxide was quantified by means of an H_2_O_2_-dependent chemiluminescence reaction (**A**). For each measurement, 200 μl of the respective supernatants were added to the cell cultures. The hydrogen peroxide formation was monitored at different time intervals upon the addition of supernatants of *C. annuum* cell wall material (**✶**), supernatants of *X. campestris* pv. campestris cultures (▲), supernatants of *X. campestris* pv. campestris cultures co-incubated with *C. annuum* cell wall material (●), and for a negative control of 200 μl water (♦). There was a clear *oxidative burst* upon the addition of a supernatant of *X. campestris* pv. campestris co-incubated with cell wall material, but an almost similar explicit reaction when a supernatant of *X. campestris* pv. campestris was added that had not been co-incubated with cell wall material. (**B**) Supernatants of *X. campestris* pv. campestris cultures were treated with polymyxin B agarose to remove LPS. Then the effect of the purified supernatants on *N. tabacum* cell suspension cultures was analyzed. The formation of H_2_O_2_ was monitored upon the addition of supernatants of *X. campestris* pv. campestris cultures (▲), supernatants of *X. campestris* pv. campestris cultures co-incubated with *C. annuum* cell wall material (●), supernatants of *X. campestris* pv. campestris cultures purified from LPS (■), supernatants of *X. campestris* pv. campestris cultures co-incubated with *C. annuum* cell wall material and purified from LPS (**✶**), and after adding 200 μl water as a negative control (♦). Removing the LPS reduced the response to *X. campestris* pv. campestris supernatant to the level of the water control. In contrast to this, the removal of LPS reduced the amplitude of the cell culture response to *X. campestris* pv. campestris co-incubated with cell wall material, but this supernatant still evoked a clear *oxidative burst* reaction.

This hypothesis was tested experimentally by co-incubating the bacteria with isolated cell wall material. Plant cell walls were prepared from *C. annuum* leafs detached from 6 week old plants grown in the greenhouse. The plant material was homogenized and extracted with aqueous and organic solvent systems, resulting in a crude cell wall preparation. This cell wall material was incubated for 12 h with *X. campestris* pv. campestris B100-Bac2 cells. The incubation was carried out in phosphate buffer to avoid interference by any additional nutrient source for the bacteria. After removing cell wall fragments and bacteria by centrifugation, the supernatant was boiled to inactivate all enzyme activity (5 min, 100°C), centrifuged again and dialyzed with a molecular-weight cut-off of 1000 Da. These samples were tested for elicitor activity in tobacco suspension cell cultures by measuring H_2_O_2_ generation, the so-called *oxidative burst*. While the supernatant of incubated cell walls induced no activity, the cell walls co-incubated with *X. campestris* pv. campestris gave rise to an *oxidative burst* that indicated the presence of a soluble elicitor (Figure 4A). All experiments performed to characterize the elicitor were initially carried out with plant suspension cell cultures from the non-host *N. tabacum*, since they are easier to handle and more responsive to elicitors than pepper cell cultures. Parallel controls containing just *X. campestris* pv. campestris bacteria or just cell wall material, respectively, were prepared. Unexpectedly, the *X. campestris* pv. campestris control caused an *oxidative burst* reaction with an amplitude that indicated nearly half of the activity observed in the measurement with the *X. campestris* pv. campestris-cell wall co-incubation. A possible explanation could be derived from previous experiments. It was shown that *X. campestris* pv. campestris lipopolysaccharides (LPSs) are MAMPs that induce pronounced elicitor activity [26,69]. Since LPS is also released to the supernatant and would not be removed or inactivated by the heat treatment, these molecules could be responsible for the *oxidative burst* caused by the *X. campestris* pv. campestris supernatant. To purify the supernatants from LPS, polymyxin B agarose was employed, which binds LPS with high affinity. By this method, essentially all elicitor activity could be removed from the *X. campestris* pv. campestris supernatant (Figure 4B). In contrast, for the *X. campestris* pv. campestris cell wall co-incubation, the polymyxin B agarose treatment reduced the elicitor activity only by about 50%. Obviously, a heat-stable elicitor, differing from bacterial LPS, had remained within this sample.

**Figure 5:**
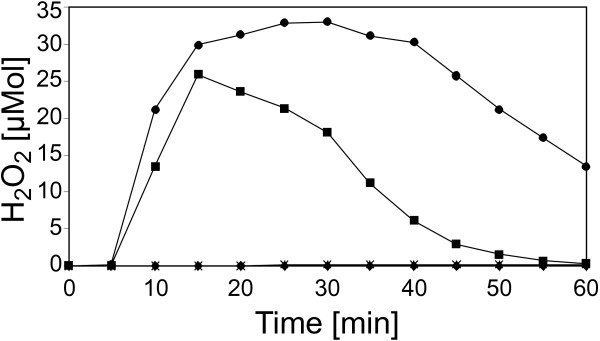
**Hydrogen peroxide formation in *C. annuum *cell suspension cultures upon elicitation with supernatant of an *X. campestris *pv. campestris *exbD2 *mutant co-incubated with cell wall material.** To assess the role of the *exbD2* gene in provoking defense reactions in non-host plants, cultures of the *X. campestris* pv. campestris mutant strain B100-11.03 were co-incubated with cell wall material from *C. annuum*. Then the formation of H_2_O_2_ was monitored in cell suspension cultures of *C. annuum* upon the addition either supernatants of *X. campestris* pv. campestris wild-type cultures (●), supernatants of *X. campestris* pv. campestris cultures affected in *exbD2* that were co-incubated with *C. annuum* cell wall material (♦), invertase as a positive control (■), or *C. annuum* cell wall material employed as negative control (**✶**). The mutated bacterial mutant strain deficient in *exbD2* could not evoke an *oxidative burst* reaction.

In the *X. campestris* pv. campestris mutant strain B100-11.03, the *exbD2* gene had been inactivated [64]. While this has no effect on iron uptake [64], the main function usually associated with the TonB import system, this mutant is affected in pathogenicity on non-host plants [66] and was now shown to lack pectate lyase activity unless complemented with a constitutively expressed pectate lyase gene. Hence, it was tempting to analyze the effect of the mutant B100-11.03 on *C. annuum* suspension cell cultures. While the well-known elicitor invertase and supernatant of the wild-type *X. campestris* pv. campestris B100 caused typical *oxidative burst* reactions, there was no response to the mutant B100-11.03 (Figure 5). Thus again, an involvement of the affected *exbD2* gene in the production of the elicitor was obvious.

### Evidence that the newly formed elicitor is an oligogalacturonide DAMP

**Figure 6:**
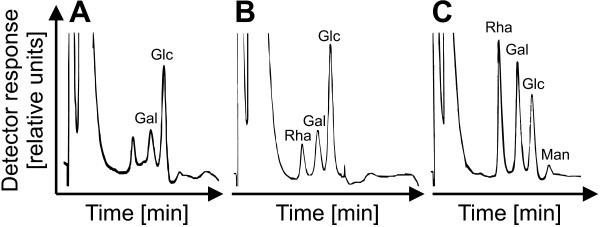
**Effect of the co-incubation of *X. campestris *pv. campestris with plant cell wall material on the composition of the dissolved monosaccharides.** The identity and relative amounts of the monosaccharides in the supernatant of *X. campestris* pv. campestris co-incubated with cell wall material of *C. annuum* was determined by HPAEC. The sugars were separated and identified using an isocratic elution with10 mM sodium hydroxide and amperometric detection on a CarboPac® PA-100 column, a set up that allows detecting the dissolved neutral sugars. The results were compared to the supernatant of an *X. campestris* pv. campestris culture that had had no contact to plant cell wall material, and to analogously treated cell wall material that had not been incubated with bacteria. The supernatants of plant cell wall material (**A**) and the *X. campestris* pv. campestris culture (**B**), which were analyzed as controls, were both mainly composed of glucose (Glc), galactose (Gal), and rhamnose (Rha). When plant cell wall material and *X. campestris* pv. campestris culture were co-incubated (**C**), the amounts of rhamnose and galactose increased dramatically, reverting the original relative abundances. In addition, small amounts of mannose (Man) became detectable.

The isolation of the cell wall derived elicitor excluded proteins as active compound as the heating step (5 min 100°C) with subsequent centrifugation should remove or inactivate proteins from the supernatant. Considering these preliminary facts and that *X. campestris* pv. campestris is not known to produce pectate, the most likely candidate for an elicitor was an oligosaccharide or polysaccharide originating from enzymatic digestion of the plant cell wall. To further characterize the elicitor, the supernatant was treated with periodic acid, which is able to oxidize carbohydrates. This treatment led to a completely inactive supernatant that could not provoke *oxidative bursts* (data not shown). This was in good accordance with an elicitor composed of carbohydrates like oligosaccharides or polysaccharides. To further characterize the elicitor, the monosaccharide composition of the supernatant was determined by total hydrolysis with trifluoroacetic acid. The resulting monosaccharide sugars were identified by HPAEC (high-performance anion exchange chromatography; Figure 6). Glucose was particular abundant in the controls, *X. campestris* pv. campestris bacteria and plant cell wall supernatant, with minor amounts of galactose and rhamnose. In contrast, the co-incubation suspension of plant cell wall material and bacteria showed a different distribution of neutral sugars. Here, rhamnose and galactose were abundant while glucose was present in smaller amounts. The co-incubation contained also a small amount of mannose. The sugars abundant in the co-incubation suspension are constituents of plant cell walls. Rhamnose and galactose are for example components of hemi-celluloses.

**Figure 7:**
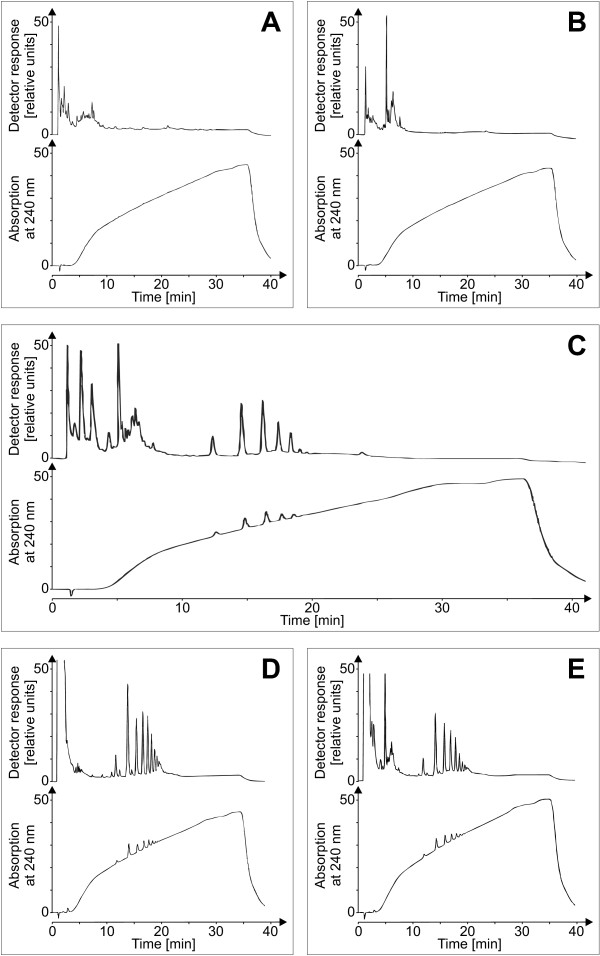
**HPAEC characterization of the elicitor-active compound.** A sodium acetate gradient ranging at 0.1 M NaOH from 0.01 M to 1 M sodium acetate with a plateau of 10 min. at a concentration of 0.7 M facilitated the identification of oligosaccharides on a CarboPac® PA-100 column with pulsed amperometric and UV-detection. Supernatants of *C. annuum* cell wall material (**A**) and an *X. campestris* pv. campestris culture (**B**) displayed no oligosaccharide signals. However, when *C. annuum* cell wall material was co-incubated with an *X. campestris* pv. campestris culture (**C**), characteristic peaks were detected that eluted between 10 min and 20 min. and that indicated the formation of oligosaccharides. A pectate standard of OGAs generated by digesting commercially available pectin with pectate lyase was analyzed as a control (**D**). The characteristic oligosaccharide peaks of both runs (**C** and **D**) were eluted at similar retention times. When the pectate standard was mixed with co-incubation supernatant, the HPAEC analysis indicated perfect overlapping of the congruent oligosaccharide peaks (**E**). Hence it was plausible to identify the oligosaccharides from the co-incubation of *C. annuum* cell wall material and *X. campestris* pv. campestris culture as OGAs.

Another major component of the plant cell wall is galacturonate, which is the building block of pectate and which in combination with rhamnose. To monitor also this compound, compositional analyses of the charged sugars were carried out using HPAE chromatography. These experiments gave evidence that the co-incubation of plant cell wall material and *X. campestris* pv. campestris contained more galacturonate than the controls (data not shown). As *Xanthomonas* has extracellular pectate lyases, it seemed reasonable that the elicitor-active compound could be a pectate fragment from the plant cell wall and hence a DAMP, as it was reported for *E. carotovora*[19]. The elicitor-active compound was analyzed via HPAE-chromatography to test this hypothesis (Figure 7). While no oligosaccharides were indicated for the individual supernatants of bacteria and cell walls, respectively, the co-incubation of both resulted in the formation of a distinct oligosaccharide pattern. The elution profile of these oligosaccharides from a gradient ranging from 0.01 M to 1 M sodium acetate indicated negatively charged oligosaccharides. Complementarily to the pulsed amperometric detection, UV-absorption was measured at 240 nm. The newly formed oligosaccharides exhibited UV-absorption. This criterion reasonably pointed to OGAs with an unsaturated C-C bond produced by lyase activity. As a standard, purified pectin was depolymerized by commercially obtained pectate lyase. The co-incubation showed the same elution profile as the depolymerized pectate standard, but a different quantitative distribution of the degrees of polymerization. Co-injection of the elicitor-active compounds with a pectate standard showed no differences between the two elution patterns, leading to the well-founded assumption that bacterial exoenzymes, most likely a bacterial lyase, were responsible for the release of these OGAs from the plant cell wall.

**Figure 8:**
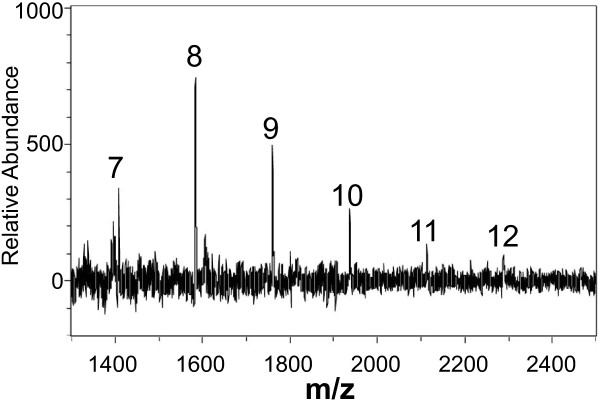
**MALDI-TOF MS of oligosaccharides released from *C. annuum *cell walls by co-incubation with *X. campestris *pv. campestris.** Cell walls of *C. annuum* and bacteria were co-incubated over night and the cell-free supernatant was desalted and lyophilized. This material was applied to MALDI-TOF MS using the negative-ion mode. A characteristic ladder of negatively charged ions was obtained. Mass differences correspond to that of OGAs of different degrees of polymerization (DP). Ions that correspond to DP 7 to 12 are indicated.

The structure of the OGA DAMP was further characterized by mass spectrometry. Upon desalting and lyophylization, the supernatant of the co-incubation of cell wall material and *X. campestris* pv. campestris was analyzed by MALDI-TOF MS (Figure 8). Mass fingerprints obtained in negative-ion mode displayed a ladder-like pattern with identical mass differences corresponding to the molecular weight of galacturonic acid. The analysis of the co-incubation revealed a prevalence of OGAs with degrees of polymerization (DP) around 8 (Figure 8). Combined with the results of total hydrolysis and monosaccharide identification by HPLC, this MALDI-TOF MS data strongly indicates the presence of linear OGAs within the supernatant of the co-incubation. Furthermore, a covalent carbon double bond can be assumed for the reducing end of the oligosaccharide due to the UV-absorption of these oligomers.

### Elicitor activity of pectate fragments in *N. tabacum* and *C. annuum* cell suspension cultures

**Figure 9:**
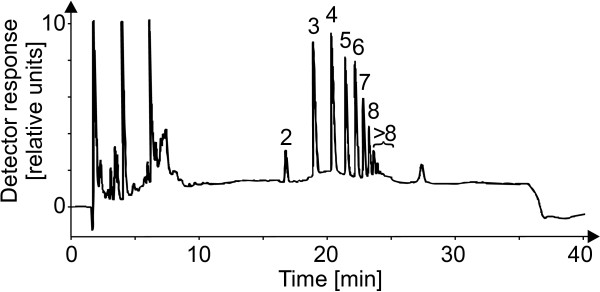
**Isolation of oligogalacturonides (OGAs) with varying degree of polymerization.** As the chromatogram of *C. annuum* cell wall material co-incubated with an *X. campestris* pv. campestris culture was identical to OGAs derived from pectin digested with a pectate lyase, the products of the co-incubation were assumed to be OGAs, too. The activity of the *X. campestris* pv. campestris culture supernatant had obviously generated a diverse set of OGAs varying by their degree of polymerization (DP), with a minimal DP of 2, see main text. To allow a further characterization of the OGAs, eluted fractions representing the different individual OGAs were isolated by a sodium acetate gradient, ranging from 0.01 M to 1 M, 0.1 M NaOH with a plateau of 10 min. at a concentration of 0.7 M on a semi-preparative CarboPac® PA-1 column.

To assess their functional roles, OGAs with different DPs were isolated. The gradient that had been employed successfully in the qualitative analyses was applied again, now with a semi-preparative column to obtain sufficient material for the subsequent characterizations (Figure 9). Pectate lyases are known to degrade pectate polymers mainly to oligosaccharides with DPs of 2, 3, and 4, while generating galacturonate monomers is uncommon these enzymes [37]. Thus the DP of the smallest detected OGA was supposed to be 2, and the peaks were numbered accordingly. Fractions containing oligosaccharides of a DP under 9 were isolated individually, while the fractions with saccharides of higher DPs were pooled. The purity of the isolates was tested by re-chromatography.

**Figure 10:**
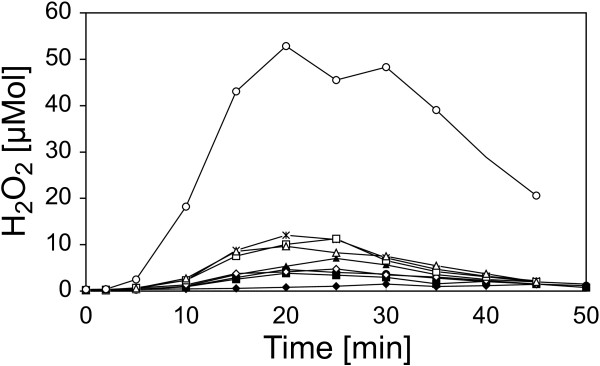
**Oxidative burst reactions in heterologous *N. tabacum *cell suspension cultures after elicitation with isolated OGAs.** To functionally characterize OGAs differing in their DPs they were checked for their capacity to evoke *oxidative burst* reactions in cell-suspension cultures of the non-host plant *N. tabacum*. Samples of the OGAs were added to the cell suspension cultures to a final concentration of 5 μg/ml. The amount of H_2_O_2_ produced upon the addition of the OGAs was monitored as described before. The addition of water used as negative control (♦) had no effect, and OGAs with a DP of 2 (■), a DP of 3 (●), a DP of 4 (▲), and a DP of 6 (◊) had only minimal effects on the *N. tabacum* suspension cells. The response to OGAs with DPs of 5 (**✶**), 7 (□), and 8 (∆) was slightly stronger but still small. But for OGAs whereof the DP exceeded 8 (○), a clear *oxidative burst* reaction was observed. This indicated the largest OGA fraction as elicitor of the non-host plant defense against *X. campestris* pv. campestris.

**Figure 11:**
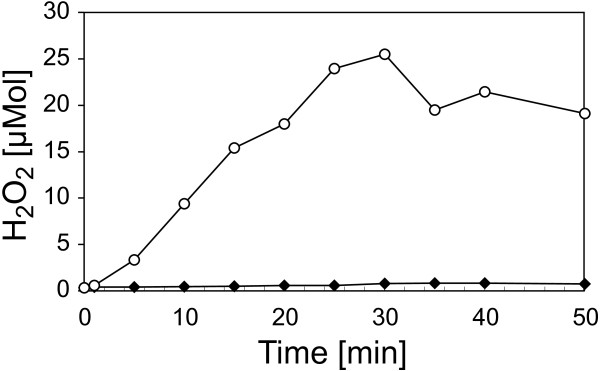
**Oxidative burst reaction in homologous *C. annuum *suspension cell cultures after elicitation with OGAs of a DP exceeding 8.** A fraction of isolated OGAs, which had a DP of at least 8, was able to elicit a strong *oxidative burst* reaction in heterologous *N. tabacum* suspension cell cultures (Figure 10). Now this OGA fraction was tested in homologous *C. annuum* suspension cell cultures. Samples were added to the *C. annuum* culture to a final concentration of 5 mg/ml (○). A negative control contained only water (♦). Once more this OGA fraction evoked a strong oxidative burst, similar to the reaction in *N. tabacum*. These observations show that OGAs with a DP of at least 8 that were generated by an *X. campestris* pv. campestris culture from co-incubated *C. annuum* cell wall material are a powerful endogenous elicitor.

The isolated OGAs were then tested for their ability to induce *oxidative burst* reactions in the heterologous non-host plant cell suspension cultures of *N. tabacum* (Figure 10). After addition of the isolates to the cell suspension cultures, small OGAs (DP 1 to 4) had only weak elicitor activities. With increasing degree of polymerization (DP 4 to 8), the elicitor activity of the isolates rose clearly. The pooled OGAs with a DP exceeding 8 were able to induce an *oxidative burst* similar to that of a general elicitor like yeast extract. As this isolate was a mixture of different DPs, the concentration of the elicitor-active OGAs was well at a nanomolar level. Finally, the response of cell suspension cultures of the homologous non-host plant *C. annuum* to the OGA elicitor (DP > 8) was measured. The cultures showed a specific *oxidative burst* reaction (Figure 11). The reaction exhibited a time course with a maximum at 25 to 30 min. and an amplitude of 25 to 50 μmol H_2_O_2_, both comparable to the reaction observed for the cell cultures of the heterologous non-host plant tobacco. All these results confirmed the identification of OGAs as a DAMP generated by the activity of *X. campestris* pv. campestris pectate lyases.

To further verify the role of the TonB system core genes and particular *exbD2* in generating the OGA DAMP, we resumed analyzing the mutants deficient in these genes [64,66]. Cell-free supernatants of *X. campestris* pv. campestris cultivations that had been co-incubated with *C. annuum* cell wall material had been shown to induce *oxidative burst* reactions in suspension cell cultures of non-host plants (Figure 4), while the supernatant of an analogously cultivated mutant strain deficient in *exbD2* evoked no *oxidative burst* in a non-host suspension cell culture (Figure 5). Now we tested the effect of cell-free supernatants obtained from co-incubating *X. campestris* pv. campestris strains with pectin on non-host cell suspension cultures concerning their ability to induce *oxidative burst* reactions. Mutants deficient in all genes of the *X. campestris* pv. campestris TonB core system including *exbD2* were tested in this approach, and turned out to be clearly affected in evoking *oxidative burst* reactions. The *oxidative burst* reactions in non-host suspension cell cultures were recovered when the disrupted genes were complemented specifically with complete copies of the respective genes (Additional file 4). The hydrogen peroxide concentrations measured in response to aliquots of cell-free supernatants from cultivations of the complemented mutants in the presence of pectin was at least at wild-type level. This clearly underlines that the genes of the bacterial TonB core system including *exbD2* are involved in linking the bacterial response to the presence of pectin with a specific defense reaction of non-host plants.

## Discussion

Most bacterial pathogens produce a wide variety of cell wall degrading enzymes like endoglucanases, cellulases, pectinases, hemicellulases and lyases. In case of *X. campestris* pv. campestris, genes coding for almost 30 enzymes probably involved in cell-wall degradation were identified based on genome data [43,45,46]. Among them were several genes involved in degrading polygalacturonic acid (Additional file 5: Table S2). In consequence, cell wall degradation by *X. campestris* pv. campestris is assumed to result in the release of a complex mixture of poly- and oligosaccharides to the surrounding medium. It is in the best advantage of plants to recognize such signals of microbial pathogenicity as DAMPs in order to initiate suitable defense reactions. Plants are able to perceive diverse signal molecules such as the yeast elicitor in tobacco [70], bacterial flagellin [71,72], harpin proteins [5-9], Hrp proteins from *X. campestris*[31], fungal proteins in parsley [73] and fungal exoenzymes in tobacco [74]. Rouet-Mayer et al. were also able to show that fungal lyase represents a different chemical stimulus than the OGAs produced from the cell walls by this enzyme’s activity and that both these elicitors despite their common origin activated at least partially differing signal transduction pathways. The fact that tobacco is not only able to perceive the products of enzymatic digestion, but also the enzyme itself, shows how crucial it is for the plant to recognize the pathogenic fungus.

Here we report on the release of elicitor-active compounds obtained from the co-incubation of *C. annuum* cell walls with *X. campestris* pv. campestris. The co-incubation was carried out using a crude cell wall extract from pepper leafs and the *X. campestris* pv. campestris strain Bac2. The use of crude cell wall extracts instead of complete plants or leafs has the advantage that all products resulting from the incubation can originate only from the plant cell wall material or the bacteria. Orientation experiments indicated that cell wall-derived oligosaccharides were responsible for the elicitor activity. To identify the elicitor-active compound, HPAE chromatography [75] was employed. First hints on the origin of the elicitor-active molecules were obtained by analyzing the composition of neutral sugars and uronic acids. In comparison to the controls, an increased abundance of typical cell wall sugars was observed when *X. campestris* pv. campestris and cell-free pepper cell wall material were co-incubated. In the subsequent characterization of the oligosaccharide composition using HPAEC [76], UV absorption was measured in addition to the PAD signal in order to detect double-bonds in the newly formed oligosaccharides. This resulted in identifying the elicitor-active compounds as pectin fragments with a varying degree of polymerization (DP) by comparing the elution profile to a standard derived from pectin digested by a pectate lyase from a commercially supplier. MALDI-TOF MS was used as a valuable tool to obtain further structural information on the isolated oligosaccharides. These fragments with different DPs were then isolated with preparative HPAEC and tested for their elicitor activities.

The highest elicitor activity was found for OGAs with a DP exceeding 8. This result corresponds well with data from Svalheim & Robertson [77], who showed that OGAs released by fungal enzymes with DPs ranging from 9 to12 are able to elicit *oxidative burst* reactions in cucumber hypocotyl segments. It also fits well with other data summarized by Ryan [78], showing that different oligosaccharides induce a vast variety of plant defense responses. For example, oligomeric fragments of chitosan with DPs ranging from 6 to 11 are able to induce defensive mechanisms in tissues of several plants. OGAs with a DP below 9 are unable to induce phytoalexin production in soybean cotyledons [20], which corresponds well with the *X. campestris* pv. campestris – pepper system, where most of the elicitor activity resides in OGAs of a DP exceeding 8.

Interestingly, OGAs can have different roles in other plant-pathogen interactions. In wheat plants, small oligomers of galacturonic acid (dimers and trimers) have a completely different function as they act as suppressors of the plant pathogen defense and thereby promote the growth of pathogenic fungi [76]. In *A. thaliana*, where WAK1 was recently identified as OGA receptor [21,23], only small cell wall-derived OGAs with DPs of 2 to 6 have been reported to induce genes involved in the plant response to cell wall-degrading enzymes from the pathogen *E. carotovora*[79].

Plants need to permanently monitor whether there are indications for pathogen attack, a task that is not trivial as it requires to efficiently filter pathogen-related signals from others, like those generated by commensal or symbiotic microorganism. For each plant it is of fundamental importance to decide correctly whether to initiate defense or not, as defense includes expensive measures like sacrificing plant tissue by intentional cell death at the assumed infection site, while mistakenly omitted defense can be lethal [80]. Analyzing the interaction of pathogens with non-host plants is an approach to identify the molecular nature of plant-pathogen interactions. Beside the highly specific recognition of *avr* gene products interactions with host plants [81], lipopolysaccharides [26,27], muropeptides [30], *hrp* gene products [31], secreted proteins [82] and the pectate-derived DAMP described in this study contribute to the reaction of non-host cells in response to *Xanthomonas*. Obviously, all these MAMPs and DAMPs are part of the very complex and specific damage- and microbe-associated molecular signal, where individual elicitors contribute in a complex manner [83] to obtain an optimal decision of the plant whether to initiate defense with all its costly consequences or not.

While the *A. thaliana* OGA receptor WAK1 was recently identified [21,23], it is now fascinating to see that the generation of a DAMP similar to that perceived by WAK1 is related to bacterial trans-envelope signaling. Recently, trans-envelope signaling has been shown to be a second important function of TonB systems [61,84] besides the uptake of nutrients. A model describing this signaling mechanism assumes that members of a specific subgroup of the TonB-dependent receptors, which share a common N-terminal extension and which were termed TonB-dependent transducers, perceive an environmental signal in the outer membrane [84]. Such TonB-dependent transducers are energized via the TonB-ExbB-ExbD core complex, while their N-terminal extension permits contacting periplasmic structures of anti-sigma factors that are localized in the inner membrane. The anti-sigma factors can then interact with ECF family sigma factors [84,85], which can modulate bacterial gene expression at the transcriptional level. Probably the best understood paradigm for TonB-dependent trans-envelope signaling is the Fec signaling pathway of *E. coli*[61]. The *exbD2* gene product of *X. campestris* pv. campestris B100 seems involved in trans-envelope signaling via the TonB system, while the *exbD1* gene is also required to import substances like ferric iron [64]. However the situation could be more complex, as *exbD2* might also be involved in uptake of cell wall degradation products, and as *exbD1* might be involved in further so far unidentified signaling processes. Currently there is no evidence that the products of both genes are involved in both functions, transportation and signaling. But likewise, so far there is no reason to assume strict task sharing, where the *exbD1* gene product is exclusively required for transport, while ExbD2 is specialized on signaling.

Further research could shed more light on the processes involved in bacterial reaction to the presence of pectin. Obviously, extracellular pectin-degrading enzymes are induced. But it is completely unclear which mechanisms are involved, and what kind of role the TonB core system plays. It could be just involved in importing polygalacturonic acid or derivatives of it. Imported galacturonic acid compounds could be perceived by an intracellular factor like a transcriptional regulator. Alternatively, the TonB system could be directly involved in signaling via an anti-sigma factor as described by Koebnik [84]. Further more, there is no reason to exclude regulatory processes at post-transcriptional levels. Likewise, the specific roles of the enzymes involved in pectin degradation are unclear. The genome of *X. campestris* pv. campestris B100 includes six genes of enzymes that cleave the glycosidic bonds between adjacent glucuronic acid residues (Additional file 5: Table S2). The product of the polygalacturonase gene *pglA2* is similar to a recently characterized *X. fastidiosa* enzyme [48], and the truncated pectate lyase encoded by *pel4* is partially similar to an enzyme from *Pseudomonas cellulosa*[86], but seemed to lack the carbohydrate-binding module (CBM) [87] of the *P. cellulosa* enzyme. A polygalacturonate-induced gene for an X. campestris pv. campestris TonB-dependent receptor like two neighboring genes encoding degrading enzymes [62] are promising candidates for being involved in the response to the presence of polygalacturonate. However, like for other *Xanthomonas* enzymes that degrade plant cell-wall constituents, the kinetic properties of the pectin-degrading enzymes are not known, nor is there evidence for the regulation and expression of their genes or for regulatory processes that directly address the enzymes.

## Conclusions

As far as we know, we report here for the first time on a DAMP that is produced by *Xanthomonas* exoenzymes from non-host plant cell walls. With the characterization of a DAMP produced by *X. campestris* pv. campestris, which was identified as an OGA, we were able to identify a further component of the complex network of signals that determines whether a plant is a host for *X. campestris* pv. campestris or whether it is resistant to this pathogen. So far, DAMPs were mainly known to be generated by fungal pathogens [17-20], and so far there are rather few examples where the signaling mechanisms have been analyzed profoundly at a molecular level. Due to the reduced complexity of prokaryotes, spending more effort on analyzing bacteria-generated DAMPs may also be a promising complement to studying fungi-based systems for pragmatic reasons, as experiments may be simpler in design, with the additional perspective of utilizing results provided by high-throughput approaches in the genomics and post-genomics disciplines for many bacteria.

**Figure 12:**
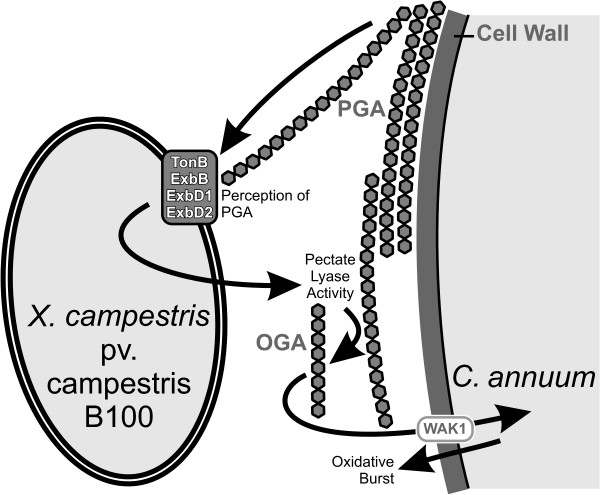
**Schematic overview on the interactions of *X. campestris *pv. campestris and *C. annuum *analyzed in this work.** A major plant cell wall component is pectate, a polygalacturonide (PGA). Pectate is perceived by *X. campestris* pv. campestris by means of the TonB system. ExbD2, which is not required for ferric iron uptake, is essential for this process. This induces extracellular pectate lyase activity, resulting in the generation of OGAs. Extracellular OGAs consisting of at least 8 galacturonate residues are recognized by *C. annuum* as a DAMP, resulting in the initiation of defensive measures like an *oxidative burst* reaction. The presence of a PRR similar to WAK1 is supposed for *C. annuum*. WAK1 has been identified recently in *A. thaliana* as a receptor that specifically perceives OGAs [23].

This work gives plausible evidence that ExbD2 is involved in transducing information on the presence of plant cell wall-derived material in the bacterial environment to the interior of the bacterial cell, leading to bacterial pectate lyase activity in the extracellular medium, which in return provokes the defense of non-host plants that can be monitored by measuring the *oxidative burst* reaction (Figure 12). Thus, the *exbD2* gene product seems involved in trans-envelope signaling via the TonB system.

Against the emerging background of TonB-related signal transduction [84] it is not too surprising to see an isoform of ExbD being involved in signaling. Nevertheless, the experimental results that indicate the involvement of ExbD2 in transducing a plant cell-wall-derived signal raise the question whether the *E. coli* paradigm on tonB functionality needs to be adapted or extended for *X. campestris* pv. campestris, as in *E. coli* ExbD (like ExbB) is supposed to be involved in signaling exclusively by contributing to energizing the outer membrane TonB-dependent transducer via TonB. The specific involvement of ExbD2 in signaling may indicate a more direct role of this ExbD isoform in signal transduction.

## Methods

### Cultivation of *Xanthomonas campestris* pv. campestris

The bacterial strains and plasmids used in this study are listed in Table 1. Unless otherwise stated, *X. campestris* pv. campestris was grown at 30°C on solid TY medium (5 g tryptone, 3 g yeast extract, 0.4 g CaCl_2_, The bacterial strains and plasmids used in this study are listed in Table 1, 12 g agar, per l), for strain B100-Bac2 supplemented with 150 mg bacitracin per l. For the *X. campestris* pv. campestris strains B100-5.05, B100-7.03, and B100-9.01, the medium was supplemented with FeSO_4_ to a final concentration of 100 mM as described previously [64]. Alternatively, bacteria were grown in modified liquid M9 minimal medium supplemented with 0.05% casamino acids [88]. Unless otherwise specified, minimal medium was supplemented with glucose or polygalacturonic acid at final concentrations of 2% or 0.25%, respectively. Streptomycin, kanamycin, gentamicin, and chloramphenicol were added to the media when appropriate in concentrations of 800 mg per l, 80 mg per l, 20 mg per l, and 100 mg per l, respectively.

**Table 1:** Bacterial strains and plasmids used in this study

**Strain or plasmid**	**Relevant genotype and/or description**	**Source or reference**
***X. campestris*pv. campestris strains**		
B100	Wild-type, Sm^r^	[46]
B100-6.01	Control strain, carrying ΩKm(*cat*) in intergenic region flanked by *tonB1* and *exbB1*, Sm^r^, Km^r^	[64]
B100-5.05	*tonB1*-deficient mutant, Sm^r^, Km^r^	[64]
B100-7.03	*exbB1*-deficient mutant, Sm^r^, Km^r^	[64]
B100-9.01	*exbD1*-deficient mutant, Sm^r^, Km^r^	[64]
B100-11.03	*exbD2*-deficient mutant, Sm^r^, Km^r^	[64]
B100-Bac2	Bacitracin-resistant spontaneous mutant of B100, unable to produce polysaccharides, Sm^r^	D. Steinmann, CeBiTec culture collection
***E. coli*strain**		
XL1Blue	*recA1*, *thi*, *supE*44, *lac*, [F’*proAB lacI*^q^, *lacZ*ΔM15, Tn*10*(Tc^r^)]	[89]
**Plasmids**		
pUC6S	*lacZ*α, Ap^r^	[90]
pBCKS+	pUC19, *lacZ*, Cm^r^	Stratagene
pBCSK+	pUC19, *lacZ*, Cm^r^	Stratagene
pMS246	pSVB30, *aacC1*, Gm^r^	[91]
pHGW31	pHIP, *aacC1*Δ*Bgl*II, Gm^r^	[64]
pHGW241	pHGW31, *tonB1*, Gm^r^	[64]
pHGW242	pHGW31, *exbB1*, Gm^r^	[64]
pHGW243	pHGW31, *exbD1*, Gm^r^	[64]
pHGW244	pHGW31, *exbD2*, Gm^r^	[66]
pIJ3051	pLAFRI-based cosmid carrying 27.9 kb chromosomal *Bam*HI fragment of *X. campestris* pv. campestris 8004 with *pglI*, Tc^r^	[39]
pHGW260	pHGW31, 11.1 kb chromosomal *Bam*HI fragment of *X. campestris* pv. campestris 8004 with *pglI*, Gm^r^	This study
pHGW261	pBCKS+, 3.8 kb *Bam*HI-*Cla*I subfragment with *pglI* from pHGW260, Cm^r^	This study
pHGW262	pBCSK+, 3.8 kb *Bam*HI-*Cla*I subfragment with *pglI* from pHGW260, Cm^r^	This study
pHGW267	pUC6S, 3.8 kb *Bam*HI-*Cla*I subfragment with *pglI* from pHGW260 expressed from *aacC1* P_out_ promoter, Ap^r^, Gm^r^	This study

### Cultivation of *C. annuum* plants

*C. annuum* (cultivar California Wonder) plants derived from seedlings were grown in the greenhouse at 21°C with 12/12 day/night hours. Cell wall material was isolated from 6 weeks old plants.

### Analysis of enzyme activity

Extracellular pectate lyase activity was monitored by an agar plate test and quantified in a photometric assay [38]. For the pectate lyase assay, *X. campestris* pv. campestris cultures were grown for 24 h in M9 medium supplemented with pectate and FeSO_4_. The resulting values were calibrated to the activity of glucose-6-phosphate dehydrogenase. For the tests on agar plates [92], *X. campestris* pv. campestris strains were cultivated for 2 days on M9 medium supplemented with pectate and FeSO_4_ as described elsewhere [93].

### Genome analysis and recombinant DNA procedures

Genome data were analyzed and visualized by means of the GenDB annotation system [94]. The EDGAR software [95] was employed to compare complete *Xanthomonas* genomes that were available from public databases [42,43,45,46,96-99]. For the analysis of genes encoding polysaccharide-degrading enzymes, information provided by the CAZy database (http://www.cazy.org/) has been considered [100].

All cloning was performed applying standard methods [101] and as described previously [64,66]. An 11.1 kb chromosomal *Bam*HI fragment of *X. campestris* pv. campestris 8004 carrying the *pglI* gene in cosmid pIJ3051 [39] was inserted into the plasmid vector pHGW31 to obtain plasmid pHGW260. A 3.8 kb *Bam*HI-*Cla*I sub-fragment with the *pglI* gene was then transferred to the cloning vectors pBCKS+ and pBCSK+, resulting in the plasmids pHGW261 and pHGW262, respectively. In pHGW262, *pglI* was constitutively expressed in *E. coli* from the *lac* promoter of the pBCSK+ multiple cloning site. To express *pglI* also in *X. campestris* pv. campestris, pHGW267 was constructed by cloning the 3.8 kb *Bam*HI-*Cla*I sub-fragment with the *X. campestris* pv. campestris 8004 *pglI* gene into the multiple cloning site of pUC6S (Ap^r^) [90], where it was under the control of the constitutive P_out_ promoter of the *aacC1* gene from pMS246 [91], which was cloned as a 1 kb *Bam*HI fragment into the *Bam*HI site upstream of *pglI*.

### Isolation of plant cell wall material

Leafs of *C. annuum* were employed to obtain cell wall material. Leafs (30 g) were homogenized in 150 ml sodium acetate (50mM, pH 5) for 3 min and filtered with a fluted filter. After the filtration, the cell wall material was washed with 1 l sodium acetate (4°C), 1 l ethanol (4°C) and with 1 l acetone (−20°C). The washed material was then air dried at room temperature and stored under inert atmosphere at -20°C.

### Co-incubation of *X. campestris* pv. campestris and *C. annuum* cell wall material

5 ml *X. campestris* pv. campestris over-night liquid culture was centrifuged. After removal of the supernatant, the sediment was re-suspended in 5 ml phosphate buffer (50 mM potassium phosphate, pH 7.9). 100 mg of the isolated cell wall material was then added to this solution and incubated over night at 28°C. The sample was then centrifuged and the pellet discarded. After heating (5 min; 100°C), centrifugation (10 min 10,000×g) and dialysis (molecular weight cut off 1000), the sample was freeze-dried. Resuspended lyophilized material was then used for further experiments.

### Removing LPS from the samples via polymyxin B agarose

*X. campestris* pv. campestris lipopolysaccharides (LPSs) were removed from the elicitor preparation using a batch technique by adding an excess amount of polymyxin B agarose [102] as described in [103]. Upon addition of polymyxin B agarose (Sigma-Aldrich), the samples were shaken and centrifuged. While the pellet probably containing LPS bound to polymyxin B agarose was discarded, the supernatant was used for further analyses.

### Identification, isolation and characterization of oligosaccharides

The analyses of oligosaccharides was performed by HPAEC using a DIONEX GP-40 gradient pump; a Merck-Hitachi D-2000 Chromato Integrator; a DIONEX pulsed amperometric detector and a DIONEX UV detector.

Monosaccharide composition of isolated oligosaccharides was analyzed upon acid hydrolysis in trifluoroacetic acid (2 M; 120°C for 2 h). Neutral sugars were separated and identified using an isocratic elution (10 mM sodium hydroxide; flow 1 ml/min) with amperometric detection on a CarboPac® PA-100 column. For charged sugars a linear sodium acetate gradient ranging from 0.02 M to 0.5 M under alkaline conditions (0.1 M NaOH) with a flow rate of 1 ml/min was used [75].

Pectate fragments were separated using a sodium acetate gradient (ranging from 0.01 M to 1.0 M with a plateau of 10 min. at a concentration 0.7 M sodium acetate; 0.1 M NaOH; CarboPac® PA-100 column; flow 1 ml/min). For the identification of pectate fragments a pectate standard was generated by digestion of pectin (Pectin esterified, Sigma P-9561) by pectate lyase (Sigma P-7052). The isolation of pectate fragments was carried out under the conditions described above, but a semi-preparative column (CarboPac® PA-1; flow 2.5 ml/min) was used.

### MALDI-TOF MS of isolated oligosaccharides

Crude extracts were analyzed on a Bruker ultraflex I MALDI-TOF mass spectrometer (Bruker-Daltonics, Bremen, Germany) in the negative–ion mode. Samples were analyzed in the linear and in the reflector TOF. Gentisic acid was used as matrix. For sample preparation, 1 μl saturated gentisic acid solution was mixed with 1 μl of 50 mg ml^–1^ crude extract lyophilisate dissolved in demineralized water. One microliter of this mixture was dropped onto the MALDI target.

### Determination of the oxidative burst reaction in plant cell suspension cultures

The detection of the *oxidative burst* was performed using the H_2_O_2_-dependent chemiluminescence reaction described by Warm [104]. Three to five days after sub-cultivation 2 g of cell material from the cell suspension cultures was diluted in 8 ml of pre-incubation medium (3% w/v sucrose in 0.04 × MS [105]) and incubated for 3 to 4 hours. For the measurement of the *oxidative burst* 200 μl aliquots of these suspensions were mixed with phosphate buffer (50 mM potassium phosphate, pH 7.9) and 1.2 mM luminol in the same phosphate buffer. The reaction was started by the addition of 100 μl of 14 mM potassium hexacyanate. The luminescence was measured with a Luminometer 1250 (BioOrbit, Turku, Finland). The intensity of luminescence was calibrated for hydrogen peroxide concentrations of 0.01 mM, to 0.05 mM.

### Chemicals

Polygalacturonic acid (sodium salt), pectin and polymyxin B agarose was from Sigma-Aldrich, Taufkirchen, Germany. Unless otherwise specified, other chemicals were obtained from Merck, Darmstadt, Germany.

## Abbreviations

Ap: Ampicillin; bp: Base pair; CBM: Carbohydrate-binding Module; CDS: Protein-coding Sequence; Cm: Chloramphenicol; DAMP: Damage-associated Molecular Pattern; DF: Diffusible Factor; DP: Degree of Polymerization; DSF: Diffusible Signal Factor; Gal: Galactose; Glc: Glucose; Gm: Gentamicin; HPAE: High-performance Anion Exchange; HPAEC: High-performance Anion Exchange Chromatography; HPLC: High-performance Liquid Chromatography; HR: Hypersensitive Response; Kb: Kilobase; Km: Kanamycin; LPS: Lipopolysaccharide; MALDI-TOF: Matrix-assisted Laser Desorption/ionization time-of-flight; MAMP: Microbe-associated Molecular pattern; min: Minute(s); MS: Mass Spectroscopy; OGA: Oligogalacturonide; PAMP: Pathogen-associated Molecular Pattern; PGA: Polygalacturonide; PRR: Pattern Recognition Receptor; PTI: PAMP-triggered Immunity; r: Resistant; Rha: Rhamnose; ROS: Reactive Oxygen Species; Sm: Streptomycin; UV: Ultraviolet.

## Competing interests

The authors declare that they have no competing interests.

## Authors’ contributions

FJV has performed genomic analyses, compiled the experimental results, and wrote the main part of the manuscript. HGW initially suggested the study, provided genetic constructs, and analyzed the pectate lyase activity and its effect on the HR of *X. campestris* pv. campestris strains on *C. annuum*. HS carried out in large part the OGA-related analyses and composed an early draft of the manuscript. VKS characterized the isolated pectate fragments by HPAE chromatography. KM carried out oxidative burst measurements with suspension cell cultures of the non-host plant *N. tabacum*. HK supervised experiments carried out by HS. AP provided infrastructure and advice, in particular related to the genes of the TonB system. KN supervised the whole project, and provided part of the manuscript’s discussion section. All authors read and approved the final manuscript.

## Supplementary Material

Additional file 1 **Multiple alignment of *Xanthomonas exbD2 *gene products.** Click here for file

Additional file 2 **Figure displaying the recovery of extracellular pectate lyase activities in complemented *X. campestris *pv. campestris strains originally deficient in genes of the TonB system.** Click here for file

Additional file 3 **Table S1 with pectate lyase activity in *X. campestris *pv. campestris and *E. coli *strains.** Click here for file

Additional file 4 **Figure displaying oxidative burst reactions in heterologous *N. tabacum *cell suspension cultures upon elicitation with supernatants of *X. campestris *pv. campestris cultures deficient in genes of the TonB system.** Click here for file

Additional file 5 **Table S2 with genes of pectin-degrading enzymes in *X. campestris *pv. campestris B100.** Click here for file
