# Supplementary material for: Involvement of bacterial TonB-dependent signaling in the generation of an oligogalacturonide damage-associated molecular pattern from plant cell walls exposed to Xanthomonas campestris pv. campestris pectate lyases
Source: BMC Microbiol. 2012 Oct 19;12:239. doi: 10.1186/1471-2180-12-239 (PMC3551730; doi:10.1186/1471-2180-12-239)
Supplement: Additional file 1 — Multiple alignment of Xanthomonas exbD2 gene products. [file 1471-2180-12-239-S1.pdf]

Additional file 1 - Multiple alignment of *Xanthomonas exbd2* gene products

```
xccb100_0011      MAFSTGGNRGPMADINVTPLVDVMLVLLIIFIVTAPIMTYPIAVDLPQRVLNPPPQTTEP
XCC0011           MAFSTGGNRGPMADINVTPLVDVMLVLLIIFIVTAPIMTYPIAVDLPQRVLNPPPQTTEP
XC_0011           MAFSTGGNRGPMADINVTPLVDVMLVLLIIFIVTAPIMTYPIAVDLPQRVLNPPPQTTEP
XCR_0011          -----------MADINVTPLVDVMLVLLIIFIVTAPIMTYPIAVDLPQRVLNPPPQTTEP
XAC0011           MAFSTGGSRGPMADINVTPLVDVMLVLLIIFIVTAPIMTYPIAVDLPQRVLNPPPQTTEP
XCV0011           MAFSTGGSRGPMADINVTPLVDVMLVLLIIFIVTAPIMTYPIAVDLPQRVLNPPPQTTEP
PXO_03473         -----------MADINVTPLVDVMLVLLIIFIVTAPIMTYPIAVDLPQRVLNPPPQTTEP
XOC_0013          MAFSTGGSRGPMADINVTPLVDVMLVLLIIFIVTAPIMTYPIAVDLPQRVLNPPPQTTEP
XOO0011           MAFSTGGSRGPMADINVTPLVDVMLVLLIIFIVTAPIMTYPIAVDLPQRVLNPPPQTTEP
XOO_0011          MAFSTGGSRGPMADINVTPLVDVMLVLLIIFIVTAPIMTYPIAVDLPQRVLNPPPQTTEP
XALc_0011         MAFSSGNSRGPMADINVTPLVDVMLVLLIIFIVTAPIMTYPIDVDLPQKVINPPPQLRDP
                             ******************************* *****.*:*****  :*

xccb100_0011      PPPIELRIDASNQVFWNNSPTPVAQLQQKMEEVVQADPTNQPELRIDANEDAEYEVMAKV
XCC0011           PPPIELRIDASNQVFWNNSPTPVAQLQQKMEEVVQADPTNQPELRIDANEDAEYEVMAKV
XC_0011           PPPIELRIDASNQVFWNNSPTPVAQLQQKMEEVVQADPTNQPELRIDANEDAEYEVMAKV
XCR_0011          PPPIELRIDASNQVFWNNSPTPVAQLQQKMEEVVQADPTNQPELRIDANEDAEYEVMAKV
XAC0011           PPPIELRIDASNQVFWNNSPTPVDVLQQKMEEVVQADPTNQPELRIDANPDAEYEVMAKV
XCV0011           PPPIELRIDASNQVFWNNSPTPVDVLQQKMEEVVQADPTNQPELRIDANPDAEYEVMAKV
PXO_03473         PPPIDLRIDASNQVSWNNSPTPVDQLQQKMEEVVQADPTNQPELRIDANPDAEYEVMAKV
XOC_0013          PPPIDLRIDASNQVSWNNSPTPVDQLQQKMEEVVQADPTNQPELRIDANPDAEYEVMAKV
XOO0011           PPPIDLRIDASNQVSWNNSPTPVDQLQQKMEEVVQADPTNQPELRIDANPDAEYEVMAKV
XOO_0011          PPPIDLRIDASNQVSWNNSPTPVDQLQQKMEEVVQADPTNQPELRIDANPDAEYEVMAKV
XALc_0011         PPPIDLRIDASNQIFWNDGPVAVSALPQMMENEVQRDPTNQPELRIDANPDSEYEVMAKV
                  ****:********: **:.*..*  * * **: ** ************* *:********

xccb100_0011      LAAAKNSQMKKIGFMQ-
XCC0011           LAAAKNSQMKKIGFMQ-
XC_0011           LAAAKNSQMKKIGFMQ-
XCR_0011          LAAAKNSQMKKIGFMQ-
XAC0011           LAAAKNSQMKKIGFMQQ
XCV0011           LAAAKNSQMKKIGFMQQ
PXO_03473         LAAAKNAQMKKIGFMQQ
XOC_0013          LAAAKNAQMKKIGFMQQ
XOO0011           LAAAKNAQMKKIGFMQQ
XOO_0011          LAAAKNAQMKKIGFMQQ
XALc_0011         LAAAKNSGMKKIGFVQQ
                  ******: ******:*
```

MUSCLE (3.7) multiple sequence alignment, provided by means of the EDGAR software (Blom J, Albaum SP, Doppmeier D, Pühler A, Vorhölter FJ, Zakrzewski M, Goesmann A, 2009. EDGAR: a software framework for the comparative analysis of prokaryotic genomes. BMC Bioinformatics 10:154. PubMed PMID: 19457249).

The amino acid sequences of the exbd2 gene products are identified by their CDS IDs, as available from public databases like UniProt or KEGG.
The CDS IDs point to the following *Xanthomonas* strains.

xccb100_0011	*X. campestris* pv. campestris B100
XCC0011	*X. campestris* pv. campestris ATCC 33913
XC_0011	*X. campestris* pv. campestris 8004
XCR_0011	*X. campestris* pv. raphani 756C
XAC0011	*X. axonopodis* pv. citri 306
XCV0011	*X. campestris* pv. vesicatoria 85-10
PXO_03473	*X. oryzae* pv. oryzae PXO99A
XOC_0013	*X. oryzae* pv. oryzicola BLS256
XOO0011	*X. oryzae* pv. oryzae KACC10331
XOO_0011	*X. oryzae* pv. oryzae MAFF 311018
XALc_0011	*X. albilineans* GPE PC73
