# Supplementary material for: Involvement of bacterial TonB-dependent signaling in the generation of an oligogalacturonide damage-associated molecular pattern from plant cell walls exposed to Xanthomonas campestris pv. campestris pectate lyases
Source: BMC Microbiol. 2012 Oct 19;12:239. doi: 10.1186/1471-2180-12-239 (PMC3551730; doi:10.1186/1471-2180-12-239)
Supplement: Additional file 2 — Figure displaying the recovery of extracellular pectate lyase activities in complemented X. campestris pv. campestris strains originally deficient in genes of the TonB system. [file 1471-2180-12-239-S2.pdf]

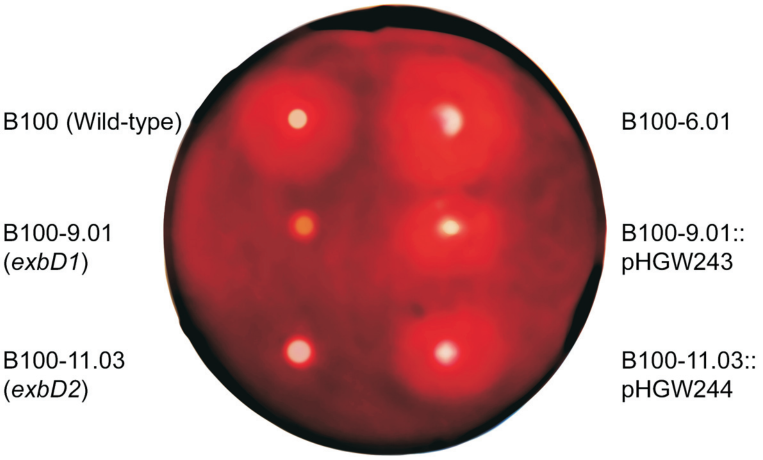

Additional file 2 - Recovery of extracellular pectate lyase activities in complemented *X. campestris* pv. campestris strains originally deficient in genes of the TonB system. The *X. campestris* pv. campestris strains were grown for two days on M9 minimal medium supplemented with polygalacturonic acid and $FeSO_4$. Staining with Ruthenium Red unveiled halos encircling inocula with extracellular pectate lyase activities. Displayed is an image of an agar plate with the wild-type strain B100 and a control strain B100-6.01 carrying an ΩKm(*cat*) insertion in the non-coding region between the *tonB* and *exbB* genes. The positions of the inocula are indicated by flanking annotations. Halos around the inocula of these strains indicate their extracellular pectate lyase activities. In the mutant strains B100-9.01 and B100-11.03 the genes *exbD1* and *exbD2*, respectively, are interrupted by ΩKm(*cat*) insertions (Wiggerich et al., 1997; Wiggerich and Pühler, 2000). Absence of halos reflects the lacking pectate lyase activities in the vicinities of the mutant inocula. Upon presence of the plasmids pHGW243 and pHGW244 that carry complete copies of the genes *exbD1* and *exbD2*, respectively (Wiggerich et al., 1997; Wiggerich and Pühler, 2000), pectate lyase activity was recovered for both mutant strains. Similar results were observed upon complementing mutant strains deficient in *tonB1* and *exbB1* (Wiggerich et al., 1997), data not shown.
