# Supplementary material for: Involvement of bacterial TonB-dependent signaling in the generation of an oligogalacturonide damage-associated molecular pattern from plant cell walls exposed to Xanthomonas campestris pv. campestris pectate lyases
Source: BMC Microbiol. 2012 Oct 19;12:239. doi: 10.1186/1471-2180-12-239 (PMC3551730; doi:10.1186/1471-2180-12-239)
Supplement: Additional file 3 — Table S1 with pectate lyase activity in X. campestris pv. campestris and E. coli strains. [file 1471-2180-12-239-S3.pdf]

Additional table 1. Pectate lyase activity of *X. campestris* pv. campestris and *E. coli* strains

Bacterial strain	PGL activity [U/ml]	Growth conditions
X. campestris pv. campestris B100 wild-type	0.96	inducing conditions (50 mg/l pectate)
X. campestris pv. campestris B100-5.05 *tonB*	< 0.01	inducing conditions (50 mg/l pectate)
X. campestris pv. campestris B100-7.03 *exbB*	< 0.01	inducing conditions (50 mg/l pectate)
X. campestris pv. campestris B100-9.01 *exbD1*	< 0.01	inducing conditions (50 mg/l pectate)
X. campestris pv. campestris B100-11.03 *exbD2*	< 0.01	inducing conditions (50 mg/l pectate)
E. coli XL1 Blue	< 0.01	non-inducing conditions (no pectate)
E. coli XL1 Blue (pHGW261)	< 0.01	non-inducing conditions (no pectate)
E. coli XL1 Blue (pHGW262)	1.35	non-inducing conditions (no pectate)
X. campestris pv. campestris B100::pHGW267	0.78	inducing conditions (50 mg/l pectate)
X. campestris pv. campestris B100::pHGW267	0.49	non-inducing conditions (no pectate)
X. campestris pv. campestris B100-11.03::pHGW267 *exbD2*	0.47	inducing conditions (50 mg/l pectate)
X. campestris pv. campestris B100-11.03::pHGW267 *exbD2*	0.45	non-inducing conditions (no pectate)

Pectate lyase activity was calibrated to the activity of glucose-6-phosphate dehydrogenase. Values below 0.01 could not be distinguished from the background.
