# Supplementary material for: Involvement of bacterial TonB-dependent signaling in the generation of an oligogalacturonide damage-associated molecular pattern from plant cell walls exposed to Xanthomonas campestris pv. campestris pectate lyases
Source: BMC Microbiol. 2012 Oct 19;12:239. doi: 10.1186/1471-2180-12-239 (PMC3551730; doi:10.1186/1471-2180-12-239)
Supplement: Additional file 4 — Figure displaying oxidative burst reactions in heterologous N. tabacum cell suspension cultures upon elicitation with supernatants of X. campestris pv. campestris cultures deficient in genes of the TonB system. [file 1471-2180-12-239-S4.pdf]

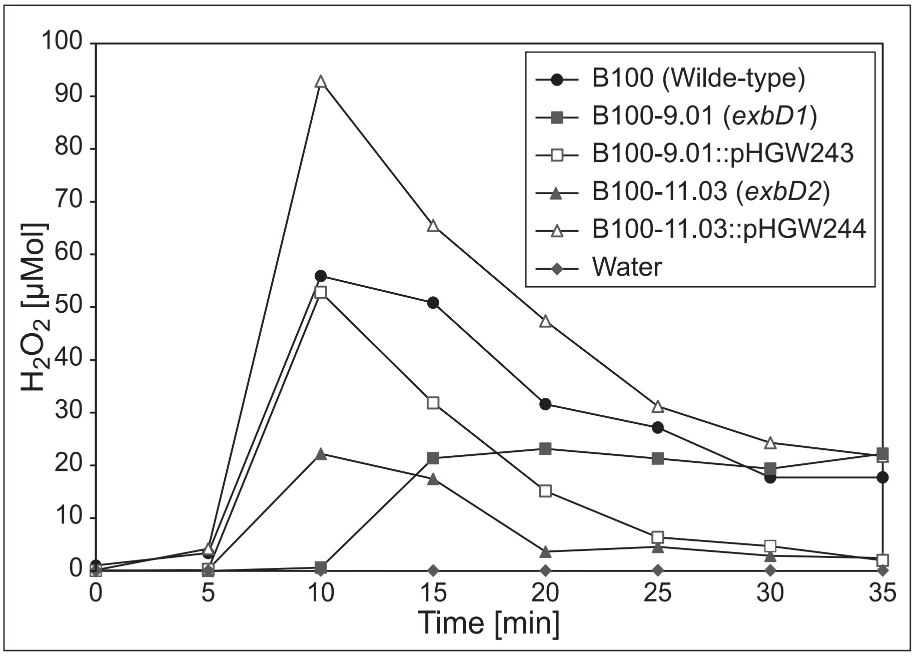

Additional file 4 - Oxidative burst reactions in heterologous *N. tabacum* cell suspension cultures upon elicitation with supernatants of *X. campestris* pv. campestris cultures deficient in genes of the TonB system. All *X. campestris* pv. campestris strains were grown in minimal medium supplemented with $FeSO_4$ and 0.25% pectin. Cell-free supernatants of these cultivations were harvested and lipopolysaccharides were removed by treatment with polymyxin B-agarose. Aliquots of the supernatants were tested in suspension cell cultures for their ability to induce the generation of hydrogen peroxide. The *X. campestris* pv. campestris strains on which the individual supernatants depended and an incubation with water as a negative control are identified in a legend on an inlay panel. Mutant strains deficient in *exbD1* and *exbD2* were complemented by means of the plasmids pHGW243 and pHGW244, respectively (Wiggerich et al., 1997; Wiggerich and Pühler, 2000). Data related to complemented mutants are indicated by open symbols. Obviously, oxidative burst reactions were recovered in the complemented strains, indicating an involvement of TonB-genes including *exbD2* in the perception of the bacteria by the plant cells.
