# Supplementary material for: Involvement of bacterial TonB-dependent signaling in the generation of an oligogalacturonide damage-associated molecular pattern from plant cell walls exposed to Xanthomonas campestris pv. campestris pectate lyases
Source: BMC Microbiol. 2012 Oct 19;12:239. doi: 10.1186/1471-2180-12-239 (PMC3551730; doi:10.1186/1471-2180-12-239)
Supplement: Additional file 5 — Table S2 with genes of pectin-degrading enzymes in X. campestris pv. campestris B100. [file 1471-2180-12-239-S5.pdf]

**Additional table 2. Genes of pectin-degrading enzymes in *X. campestris* pv. campestris B100**				
**Locus_tag**	**Name[1]**	**Gene Product**	**EC-Number**	**CAZy[2]**
Pectate lyase genes				
xcc-b100_1345	*pel1*	exported pectate lyase	4.2.2.2	PL1
xcc-b100_3710	*pel2*	exported pectate lyase	4.2.2.2	PL1
xcc-b100_3711	*pel3*	exported pectate lyase	4.2.2.2	PL1
xcc-b100_0131	*pel4*	truncated pectate lyase	4.2.2.2	PL10
Polygalacturonase genes				
xcc-b100_0738	*pglA1*	exported polygalacturonase	3.2.1.15	GH28
xcc-b100_1912	*pglA2*	exported polygalacturonase	3.2.1.15	GH28
Pectin methylesterase genes				
xcc-b100_0130	*pme1*	exported pectinesterase	3.1.1.11	CE8
xcc-b100_1913	*pme2*	exported pectinesterase	3.1.1.11	CE8

[1]) Gene name.

[2]) Enzyme family in the CAZy database (http://www.cazy.org/) for polysaccharide lyases (PL), glycoside hydrolases (GH), or carbohydrate esterases (CE).
